# Biomechanical finite element analysis of vertebral column resection and posterior unilateral vertebral resection and reconstruction osteotomy

**DOI:** 10.1186/s13018-021-02237-4

**Published:** 2021-01-28

**Authors:** Ye Han, Xiaodong Wang, Jincheng Wu, Hanpeng Xu, Zepei Zhang, Kepeng Li, Yang Song, Jun Miao

**Affiliations:** 1 Graduate School, Tianjin Medical University, Tianjin, China; 2 Department of Orthopaedics, Tianjin Hospital, No. 406, Jiefang South Road, Hexi District, Tianjin, China

**Keywords:** Old vertebral compression fracture, Biomechanics, Finite element analysis, Spinal osteotomy

## Abstract

**Background:**

Regarding the repair of vertebral compression fractures, there is a lack of adequate biomechanical verification as to whether only half of the vertebral body and the upper and lower intervertebral discs affect spinal biomechanics; there also remains debate as to the appropriate length of fixation.

**Methods:**

A model of old vertebral compression fractures with kyphosis was established based on CT data. Vertebral column resection (VCR) and posterior unilateral vertebral resection and reconstruction (PUVCR) were performed at T12; long- and short-segment fixation methods were applied, and we analyzed biomechanical changes after surgery.

**Results:**

Range of motion (ROM) decreased in all fixed models, with lumbar VCR decreasing the most and short posterior unilateral vertebral resection and reconstruction (SPUVCR) decreasing the least; in the long posterior unilateral vertebral resection and reconstruction (LPUVCR) model, the internal fixation system produced the maximum VMS stress of 213.25 mPa in a lateral bending motion and minimum stress of 40.22 mPa in a lateral bending motion in the SVCR.

**Conclusion:**

There was little difference in thoracolumbar ROM between PUVCR and VCR models, while thoracolumbar ROM was smaller in long-segment fixation than in short-segment fixation. In all models, the VMS was most significant at the screw-rod junction and greatest at the ribcage–vertebral body interface, partly explaining the high probability of internal fixation failure and prosthesis migration in these two positions.

## Introduction

Thoracolumbar vertebral compression fractures are common in orthopedics; these fractures include osteoporotic fractures in the elderly and fractures resulting from falls or car accidents. If not treated in a timely fashion, they may develop into old fractures, accompanied by kyphotic deformities. The symptoms of old vertebral fractures with kyphotic deformities include persistent low back pain, sometimes accompanied by spinal cord compression symptoms. Conservative treatment of old vertebral compression fractures with kyphotic deformities is often not effective; therefore, treatment is often surgical [1]. For severe old vertebral compression fractures with kyphotic deformities, vertebral column resection (VCR) surgery can relieve spinal cord compression and restore vector sequence balance. This has been the classical therapeutic goal. However, traditional VCR requires long operation times and substantial surgical trauma, both of which pose substantial challenges to surgeons. For these reasons, researchers proposed posterior unilateral vertebral resection and reconstruction (PUVCR) to correct kyphotic deformities using a unilateral approach and partial osteotomy. We used finite element analysis to simulate T12 VCR and PUVCR for bone cutting. Long-segment or short-segment fixation was performed to measure biomechanical stability after surgery.

Computed tomography (CT) images of normal subjects were imported into computer modeling software to establish a model, thereby simulating various surgical osteotomy modes, restoring vector balance, and installing a screw-rod system. Finally, we used finite element analysis software to calculate and compare biomechanical parameters.

## Methods

### Design, location, and timing

We performed a finite element analysis of patients treated between 1 January 2020 and 1 June 2020 at the Department of Spinal Surgery, Tianjin Hospital, Tianjin University.

### Participants

Volunteers were recruited from the Department of Spinal Surgery, Tianjin Hospital, Tianjin University. A 40-year-old man, height 176 cm, and weight 72 kg underwent spinal CT scan with three-dimensional reconstruction and MRI examination to rule out other spinal diseases. After obtaining consent from the patients and their families, informed consent forms were signed following the relevant regulations and submitted to the Ethics Committee for approval.

### Acquisition and reconstruction of CT tomography images

We obtained 128-row, 256-slice GE spiral CT thin-section scans (Sensation 16 Siemens, Germany) with a slice thickness of 1 mm and resolution of 512 × 512 to obtain sagittal two-dimensional tomographic images of the T8–L3 vertebral body, and files were saved in DICOM format.

DICOM images were imported into Mimics 20.0 (Materials Company, Leuven, Belgium) to create a 3-dimensional (3D) vertebral surface model from T10 to L2 that were stored as STL format files; 3-Matic 12.0 software (Materialise, LA, USA) was used after wrapping, smoothing, and removal of excess triangulars. Data were imported into Geomagic Studio 12.0 (Geomagic, Cary, NC, USA) for solidification.

Hypermesh (Altair Engineering, Troy, MI, USA) was used to grid and construct the intervertebral disc, bone, and ligament structures, in which the intervertebral disc was composed of annulus fibrosus matrix, nucleus pulposus, annulus fibrosus fibers, and upper and lower endplates; the nucleus pulposus accounted for 43% of the total intervertebral disc [2]. The surgical model was processed in Hypermesh, and the screw-rod system was made using Pro/Engineer PTC, MA, USA) and assembled in Hypermesh. Abaqus (Hibbitt, Karlsson, and Sorensen, Inc., Providence, RI, USA) was used for material property definition, model assembly, loading, and finite element analysis. This study’s material properties were validated based on published finite element models and thoracolumbar spines of human cadavers (Table 1) [3–5].

**Table 1:** Material properties of spine tissues used in the finite element model

The material properties of the finite element model			
Component name	Young’s modulus(MPa)	Poisson’s ratio	Cross-section area(mm^2^)
Cortical bone	12,000	0.3	-
Cancellous bone	100	0.3	-
Cartilage	10	0.4	-
Bony endplate	1000	0.4	-
Nucleus pulposus	1	0.499	-
Annulus fibrosus	450	0.3	-
ALL	20	0.3	63.7
PLL	20	0.3	20
LF	19.5	0.3	40
ISL	11.6	0.3	40
SSL	15	0.3	30
TL	58.7	0.3	3.6
CL	32.9	0.3	60

*ALL* anterior longitudinal ligament, *PLL* posterior longitudinal ligament, *ISL* interspinous ligament, *SSL* supraspinal ligament, *CL* capsular ligament, *LF* ligamantumflavum, *TL* transverse ligaments

#### Design of the kyphotic cutoff model

**Fig. 1:**
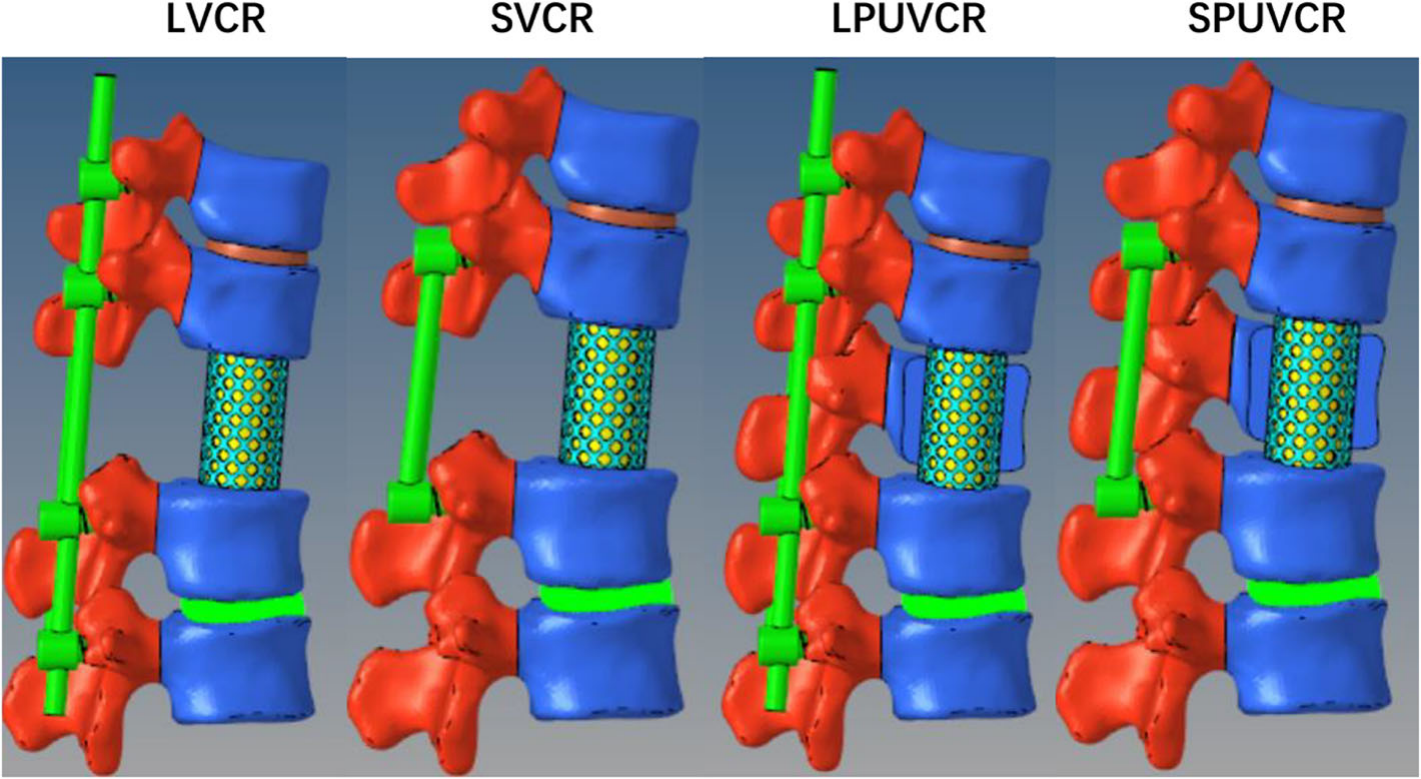
Finite element models of the fixation constructs:LVCR:long vertebral column resection, SVCR:short vertebral column resection, LPUVCR:long posterior unilateral vertebral resection and reconstruction, SPUVCR:short posterior unilateral vertebral resection and reconstruction

We fabricated four fixation models using various screw combinations. The pedicle screws inserted into T11 and 12 measured 6.0 × 40 mm, and the pedicle screws inserted into L1 and two vertebral bodies measured 6.5 × 45 mm. The LVCR model refers to the removal of the whole T12 vertebral body and T11/12, T12/L1 intervertebral disc, the application of a cylinder of 18-mm diameter and 1-mm thickness to simulate a titanium cage fixed in the vacant position of the vertebral body, with cancellous bone placed in the middle. Pedicle screw fixation was applied at T10, T11, L1, and L2. SVCR refers to the removal of pedicle screws at T10 and L2 based on LVCR. LPUVCR refers to removing T12 right vertebral plate, facet joint, and vertebral body, T11/12, T12/L1 right intervertebral disc. A cylinder with a diameter of 10 mm and a thickness of 1 mm was used to simulate the titanium cage. The cage was fixed at the vacant position of the vertebral body, and the cancellous bone is placed in the middle for testing. Pedicle screw fixation was applied at T10, T11, L1, and L2. SPUVCR refers to the removal of pedicle screws at T10 and L2 on top of LPUVCR (Fig. 1).

### Evaluate loading conditions and spinal motion simulation

Abaqus was used to assess the boundary and loading conditions and to simulate spinal motion. The L2 vertebral body was assumed to be fixed, and its substructure was set to the boundaries without displacement or rotation in all directions. Spinal motion in the sagittal, coronal, and transverse planes was defined as flexion-extension, lateral bending, and rotation, respectively. According to the human body’s bearing capacity and the previously published literature, an axial load of 200 N and an additional torque load of 7.5 Nm were applied to simulate flexion and extension, lateral bending, and rotation of the spine. An axial load was applied to the T10 vertebral body’s superior surface, and a torsional load was applied to the center of the T10 vertebral body.

### Evaluation indicators

Three metrics were used to assess the mechanical performance of the constructs: (1) ROM of the overall fixation (T10–L2); (2) von Mises stress of the instrumentation system; and (3) stress magnitude and distribution of the inferior endplate of T11 and superior endplate of L1. These measures were chosen to assess the role of various vertebral body reconstruction methods in the overall fixation system. Because only one subject was modeled, statistical analysis was not performed in this study.

## Results

### Validation of the model

The model was determined to be reasonable by comparison with biomechanical experiments and finite element models. Using the same loading conditions, the ROM values of the complete model at the moment of 7.5 N were consistent with those of previous studies [6–8]. This finding suggests that this study’s finite element model is effective for further simulation of old fractures of the thoracolumbar spine.

### ROMs for the thoracolumbar segment

**Fig. 2:**
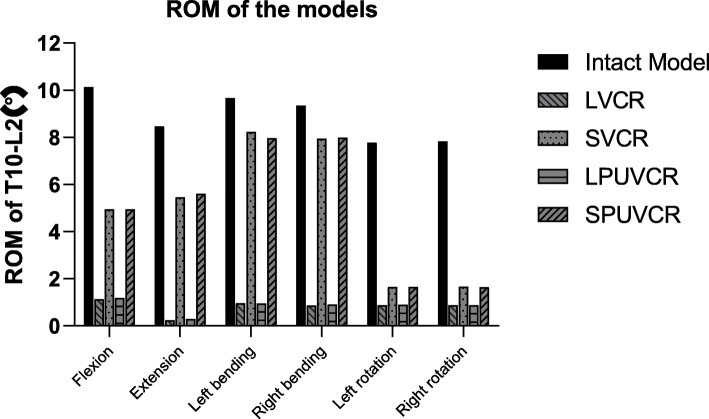
Angular rom of thoracolumbar junction (T10–L2)

Compared with the intact model, ROMs decreased in all fixed models under loading, with LVCR decreasing the most, forward flexion decreasing by 88.8%, extension decreasing by 97.3%, left lateral bending decreasing by 89.6%, right lateral bending decreasing by 90.8%, left lateral rotation decreasing by 88.8%, and right lateral rotation decreasing by 88.9%. SPUVCR decreased the least, with 51.2% decrease in forward flexion, 33.8% decrease in extension, 17.6% decrease in left lateral curve, 14.6% decrease in right lateral curve, 78.8% decrease in left rotation, and 79.1% decrease in right rotation (Fig. 2).

### Von Mises stress in the internal fixation system

**Fig. 3:**
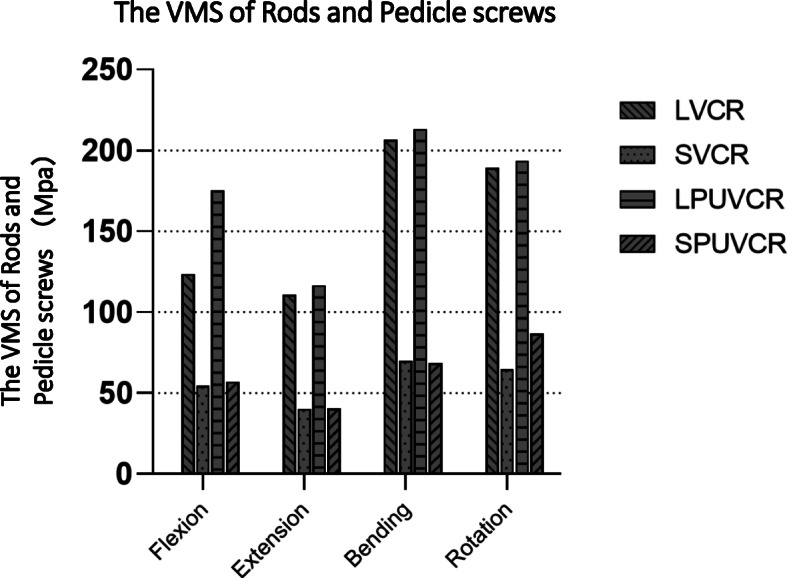
Von Mises stress on rods and pedicle screws

Regardless of the fixation mode, the screw-rod system's stress concentration point was located at the screw-rod junction position. Compared with the short-segment fixation, the stress of the screw-rod system in the long-segment fixation was within the bearing range of internal fixation, in which the maximum stress generated the lateral bending motion in the LPUVCR model, which was 213.25 mPa; the minimum stress generated the lateral bending motion in the SVCR, which was 40.22 mPa. In addition to lateral bending motion, the rod’s maximum stress was greater for PVCR than for VCR (Fig. 3).

### Stress distribution in T11 inferior endplate and L1 superior endplate

**Fig. 4:**
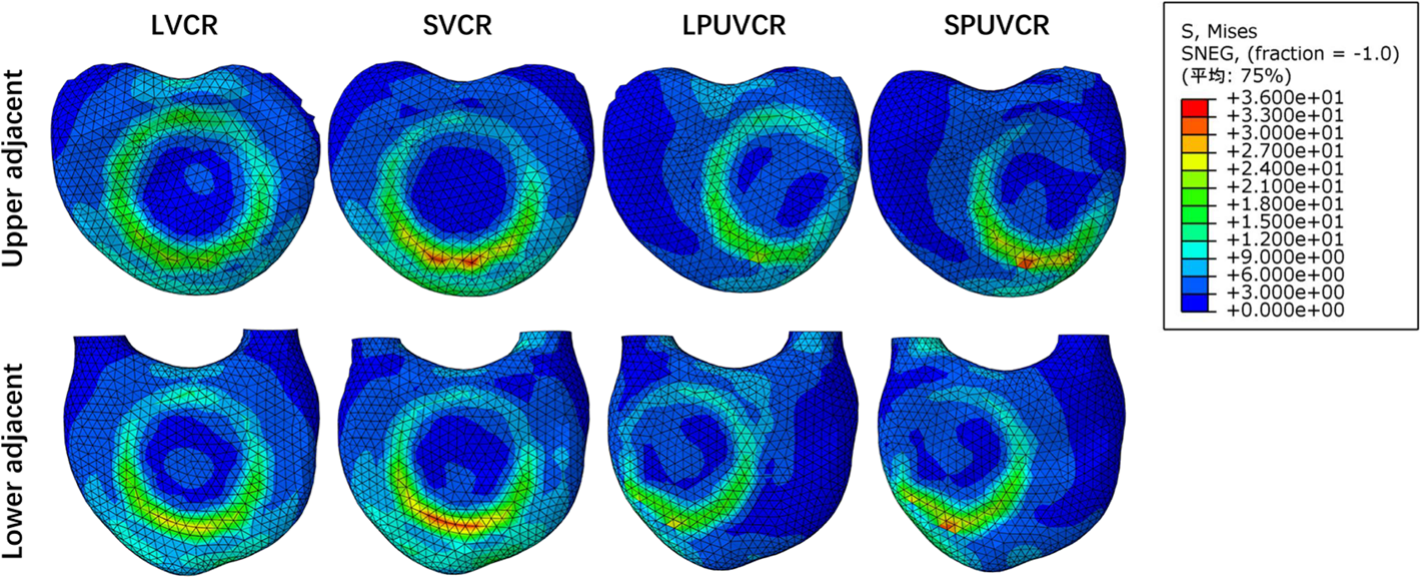
The stress in the flexion movement in the inferior endplate of T11 and the upper endplate of L1

**Fig. 5:**
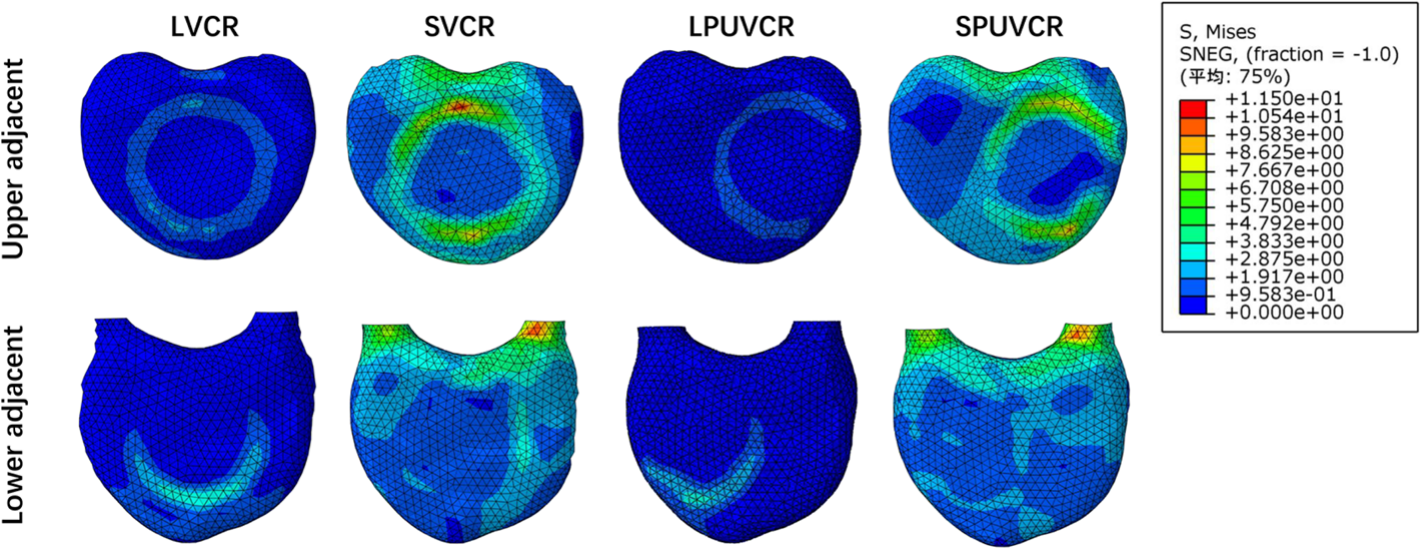
The stress in the extension movement in the inferior endplate of T11 and the upper endplate of L1

In the four models, the stress concentration area was located in the contact area between the titanium mesh and the upper and lower endplates, especially in front of the contact area between the titanium mesh and the endplates, producing more significant stress in both the upper and lower endplates. The maximum stress in the inferior endplate of T11 was produced in the lateral bending movement of the SPUVCR model, which was 36.25 mPa, while the minimum stress was produced in the extension movement of the LPVCR, which was 3.87 mPa. The minimum stress in the upper endplate of L1 was produced in the extension movement of the LPVCR, which was 1.91 mPa, while the stress was the largest in the lateral bending in the SVCR, which was 44.77 mPa. See Figs. 4 and 5 for specific stresses.

## Discussion

Compression fractures often cause old vertebral compression fractures after trauma or in elderly patients who are not diagnosed and treated in a timely fashion. The kyphotic deformity often accompanies these fractures. The clinical symptoms are primarily persistent low back pain and neurological symptoms caused by spinal cord compression; these include lower limb numbness and decreased muscle strength. When conservative treatment is ineffective, spinal cord compression can be relieved by surgery to restore the spine’s sagittal balance; this can effectively improve clinical symptoms and correct the kyphotic deformity [9–12].

VCR is a traditional procedure to treat spinal osteotomy. Through posterior resection of the spinous process vertebral plate, pedicle screws are placed on the upper and lower two vertebrae; then, through resection of the entire vertebral body and upper and lower intervertebral discs, titanium mesh is placed and fixed, followed by screw-rod fixation. VCR surgery effectively corrects severe kyphotic deformity and achieves reconstruction of the vertebral body; however, due to the difficulty of surgery, there are often problems such as longer operation time, larger amounts of blood loss, and greater degree of trauma.

PUVCR, a modified modification of VCR, exposes the vertebral body through a unilateral approach, removes half of the vertebral body and the upper and lower intervertebral discs, shortens the operation time, and reduces the blood loss while restoring the sagittal sequence of the spine [13]. Some studies suggest that PUVCR can completely decompress and effectively restore deformity in the treatment of old thoracolumbar fractures [1, 13, 14]. Nevertheless, there is a lack of effective biomechanical verification as to whether only half of the vertebral body and the upper and lower intervertebral discs affect spinal biomechanics; there also remains debate as to the appropriate length of fixation. For these reasons, in this study, we analyzed the effect of VCR and PUVCR on vertebral stability using finite element analysis.

Finite element analysis reconstructs the shape of the spine using computer simulation. It then directly interprets spinal biomechanics changes caused by internal fixation by loading its material parameters and loading conditions and guiding the surgical plan [15]. Finite element analysis can be repeatedly applied, saving medical resources while safely and efficiently simulating complicated surgical procedures. For these reasons, it has become a hot area of research.

Studies have analyzed lumbar ROM after Ponte osteotomy and showed that this technique leads to 20% instability, while total discectomy leads to further instability [16]. Due to decreased stability, additional fixation is required to enhance stability, and pedicle screw fixation is the most common fixation method in spinal fixation. Liu et al. analyzed the stress of the screw rod and found that the VMS of the pedicle screw was the smallest when the directional pedicle screw was applied (136.9 mPa), and the VMS of the pedicle screw was the largest with the hybrid application (382.6 mPa) [17]. VMS of the rod was the largest when a polyaxial pedicle screw was applied (439.9 mPa), and the VMS of the rod was the smallest when a directional pedicle screw was applied (341.7 mPa). Because most patients with old vertebral compression fractures and kyphotic deformities are elderly and have low relative bone mineral density, we applied directional pedicle screws for simulated fixation, and this can reduce the stress on the screw-rod and prevent its pullout. Natarajan et al. showed that the application of a 6-mm screw-rod system resulted in stronger fixation than a 5-mm screw-rod system, increasing the size of the system from 5 mm to 6 mm, increasing the flexion/extension moment by 8%, the torsion moment by 14%, and the lateral bending moment by 24% [18]. We applied a 6-mm screw-rod system to simulate two surgical modalities in the hope of increasing fixation strength.

Fusion fixation reduces thoracolumbar motion, as shown in previous studies [15, 19]. Lu and Lu found that the short-segment fusion fixed model decreased by 52–94% compared with the intact model [20].

There are few studies on ROM values after VCR surgery. In our test, the ROM of all models decreased relative to the intact model, with the LVCR model showing the largest decrease of 97.28% in extension and the SVCR model showing the smallest decrease of 14.90% in lateral bending. The thoracolumbar ROMs of long-segment fixed models (LVCR and LPUVCR) were significantly smaller than those of short-segment fixed models (SVCR and SPUVCR), while the ROM of the VCR model was relatively smaller than that of the PUVCR model; however, the difference was not significant. This finding suggests that the thoracolumbar segment's ROM is greatly affected by fixation; however, it has little relative relationship with the amount of vertebral body and soft tissue removed.

We found that the site with the largest VMS in internal fixation was at the screw-rod connection site in all models, explaining why internal fixation is prone to failure at the screw-rod connection site. Similar findings were reported previously [21–23]. PUVCR showed slightly higher screw and rod stresses than VCR in all loading cases except in short-segment lateral bending. In terms of titanium cages, although the maximum stress site varied in various models due to the length of fixation, the maximum value of VMS was at the contact surface site between the titanium cage and the vertebral body, suggesting that the risk of prosthesis displacement is the greatest at the contact surface between the titanium cage and the vertebral body. Compared with VCR, the PUVCR titanium cage’s stress increased by 41.9, 5.2, 3.2, and 2.2% in forward flexion, extension, lateral bending, and rotation, respectively.

In contrast, in long-segment fixation, the PUVCR titanium cage’s stress increased by 4.5%, 1.2%, and 25.2% in forward flexion, extension, and rotation, respectively, compared with VCR. In the position of the superior and inferior endplates, the stress in the short segment was significantly greater than the stress in the long segment, and this may explain why the short segment is more prone to subsidence of the anterior prosthesis. Nevertheless, the amount of vertebral body removal did not appear to affect the change of the area of stress concentration, whether it was the VCR model (LVCR, SVCR) or the PUVCR model (LPUVCR, SPUVCR). The site with higher stress was located in the contact position between the titanium cage and the endplate, suggesting that the prosthesis settlement could occur at the contact site.

When performing surgery, clinicians should pay more attention to the screw-rod junction and the contact position between the titanium cage and the endplate to prevent the fracture of the screw-rod and the settlement of the titanium cage. Simultaneously, a long-segment fixation can be applied to enhance the stability of the spine. In terms of surgical methods, both VCR and PUVCR have similar fixation strengths and can achieve adequate fixation. However, PUVCR is relatively more straightforward to operate than VCR; the operation time is shorter, and there is less blood loss.

This study has some limitations. First, we used CT scans of normal subjects to construct the old thoracolumbar fracture model, which may have impacted the test. Second, we only used one person to establish the model. Due to individual differences, there may be structural variations in vertebral bodies and ligaments. Third, our test was not verified using cadaver testing. In future studies, model reconstruction using several patients can be further performed, resulting in more objective results. The biomechanics of cadaveric specimens can also be used as a supplement to the finite element analysis.

## Conclusion

Compared with the VCR model, the thoracolumbar ROM of PUVCR was not significantly different, while the thoracolumbar ROM was less than that of short-segment fixation after long fixation. Except for short-segment rotations, the stresses were more remarkable for the PUVCR instrumentation systems than for the VCR. In the stress distribution, the screw-rod junction had the largest VMS. In contrast, the cage and vertebral body interface had the largest VMS, and this partly explained the high probability of internal fixation failure and prosthesis migration in these two positions.

## Data Availability

Please contact the corresponding author for data requests.

## Abbreviations

ROM: Range of motion
VCR: Vertebral column resection
PUVCR: Posterior unilateral vertebral resection and reconstruction
LVCR: Long vertebral column resection
SVCR: Short vertebral column resection
LPUVCR: Long posterior unilateral vertebral resection and reconstruction
SPUVCR: Short posterior unilateral vertebral resection and reconstruction
VMS: Von Mises stress
